# Screening for Potential Drug–Drug Interactions in Patients Receiving Anticoagulant Therapy: A Comparison of Three Drug Interaction Databases for Consistency in Severity Rating, Evidence Classification, and Clinical Management

**DOI:** 10.3390/medicina62050872

**Published:** 2026-05-02

**Authors:** Javedh Shareef, Sathvik B. Sridhar, Sanah Hasan, Mohammed Salim Karattuthodi

**Affiliations:** 1 Clinical Pharmacy & Pharmacology, Ras Al Khaimah College of Pharmacy, Ras Al Khaimah Medical and Health Sciences University, Ras Al Khaimah P.O. Box 11172, United Arab Emirates; 2 College of Pharmacy, Dubai Medical University, Dubai P.O. Box 20170, United Arab Emirates; 3 RAK College of Pharmacy, RAK Medical & Health Sciences University, Ras Al Khaimah P.O. Box 11172, United Arab Emirates; 4 College of Pharmacy and Health Sciences, Ajman University of Science and Technology, Ajman P.O. Box 346, United Arab Emirates; s.hasan@ajman.ac.ae; 5 Department of Pharmacy Practice, College of Pharmacy, Gulf Medical University, Ajman P.O. Box 4184, United Arab Emirates; dr.salim@gmu.ac.ae

**Keywords:** prescriptions, anticoagulants, drug interactions, treatment outcome, clinical decision-making

## Abstract

*Background and Objectives:* For patients on anticoagulants, the risk of possible drug–drug interactions (pDDIs) is particularly higher due to complex polypharmacy. Clinical decision-making is largely guided by drug interaction databases (DIDs); however, inconsistencies in programming may compromise therapeutic safety and effectiveness. The current study is designed to assess and contrast the consistency of severity rating, evidence classification, and clinical management of pDDIs across three DIDs. *Materials and Methods:* The study was conducted using real patient data from the outpatient medicine and cardiology department of a public hospital in the United Arab Emirates. Prescriptions containing anticoagulants were evaluated using three databases for pDDI screening: Micromedex, Lexicomp, and Drugs.com. Consensus was assessed using Fleiss’ kappa, and correlations between variables were evaluated using Spearman’s rank correlation coefficient, using a threshold of *p* < 0.05 to assess statistical significance. *Results:* A total of 130 prescriptions were analyzed, and 3237 pDDIs involving 1143 interaction pairs were retrieved. Of these, 107 pDDI pairs were consistently identified across all three databases. Significant inter-database variability was observed in the severity classification and management recommendations of pDDIs across the three databases. Regarding evidence classification, both Micromedex and Lexicomp rated most interactions with fair evidence, while Drugs.com provided no evidence ratings. Although some correlations were observed—particularly between Lexicomp’s and Drugs.com—overall agreement across databases was slight to fair (*p* < 0.05). *Conclusions:* Marked inconsistencies across the databases were identified in the classification and categorization of pDDIs and their associated parameters. Category-wise agreement analysis provides more meaningful insights beyond overall agreement by revealing clinically relevant concordance and divergence among databases.

## 1. Introduction

Patients on anticoagulant therapy for cardiovascular and thromboembolic disorders exhibit a greater likelihood of experiencing potential drug–drug interactions (pDDIs) [1]. There are benefits as well as risks, and vigilant clinical watch is important for bleeding or treatment failure [2,3].

High-risk groups, such as the elderly and those suffering from polymorbidities with cardiovascular and thromboembolic disorders, are commonly treated with multiple medications (polypharmacy). This increases the risk of significant pDDIs, which leads to either discordant outcomes or loss of safety and efficacy of anticoagulant therapy [4]. The simultaneous use of anticoagulants with cardiovascular, endocrine, anti-infective, neurologic, and psychiatric agents increases the likelihood of DDIs, which alter the drugs’ metabolism and/or therapeutic effects [2]. Changes in drug metabolism (by either inhibition or activation of the CYP450 system), competition for elimination (through renal or hepatic pathways), changes in drug distribution (by displacement of the drug from plasma proteins), and additive or synergistic effects in drug action (for example, increased risk of bleeding) present numerous potential pathways for pDDI. Given the need to ensure patient safety and derive the intended therapeutic benefits, systematic checking for pDDI among the prescriptions is an essential element of pDDI therapy management [5].

Incorporating drug interaction databases (DIDs) into clinical decision support systems (CDSS) can help prescribers identify and manage pDDIs in clinical practice. Optimizing patient drug therapy can be achieved by understanding the nature and clinical significance of drug interactions, as well as the severity and the level of evidence and management options provided by these databases [6,7]. The major issue affecting clinical decision-making, however, is the lack of agreement and the inconsistencies among these databases [8].

Discrepancies in the analyses of subscription-based databases against public drug information databases have been reported in studies conducted in various countries [9,10,11]. These differences may be the result of different methods of evaluating the evidence, different evaluations of the clinical importance of the interactions, and different rates of updating the databases. Inconsistencies in evidence grading and management recommendations may result in clinical uncertainty, unpredictable patient outcomes, and, more importantly, encourage the possibility of ineffective management of pDDIs [8]. This is highly pertinent in the management of anticoagulant therapy, where inadequate management of pDDIs can result in detrimental effects on patient health, including an increased risk of bleeding [12].

To date, there has been no significant research into the comparability of evidence, grading, and management recommendations of pDDIs involving anticoagulant therapies in DIDs [13]. Even fewer studies have attempted to analyze the existing real-world data in secondary care environments, where there is no uniform decision-support system and where employees rely on clinical judgment to determine the actions required to address the possible drug interactions [14]. For these reasons, this study focused on evaluating the interface of DIDs in assessing the pDDIs involving anticoagulant therapies in relation to evidence and recommendations, as well as management and severity, to determine what is available. It is intended that the findings will clarify the reliability, dependability, and consistency of various databases and inform the decision-making processes in clinical practice.

## 2. Materials and Methods

### 2.1. Study Design and Study Setting

A cross-sectional observational study was undertaken in the outpatient medicine and cardiology department for six months (Jan 2023–June 2023) in a secondary care hospital in Ras Al Khaimah, U.A.E.

The study was conducted in accordance with the Declaration of Helsinki and approved by the institutional [RAKMHSU-REC-190-2020-F-P dated 26 January 2020] and the Regional Research and Ethics Committee of Ras Al Khaimah, UAE [MOHAP/REC/2022/47-2022-F-P dated 21 February 2023].

### 2.2. Study Design

This research investigated 130 prescriptions that involve anticoagulants. It compared three databases— Micromedex® (IBM Corporation, Armonk, NY, USA) (MM), Lexicomp® (Wolters Kluwer, Hudson, OH, USA) (LC), and Drugs.com (Drug site Trust, Auckland, New Zealand) (DC) for their output and consistency of pDDIs concerning their rating of severity, documentation of quality of evidence, and clinical management suggestions for handling pDDIs.

### 2.3. Criteria for Inclusion and Exclusion

Patients aged ≥18 years visiting the outpatient medicine and cardiology department at a public hospital in the UAE, who had at least one anticoagulant prescribed, and were willing to participate, were included. Patients receiving only anticoagulant therapy or with incomplete medical records were excluded.

The study considered all drugs containing anticoagulants as study-inclusion drugs. However, during the study period, only oral anticoagulants were identified, and there were no patients undergoing treatment with parenteral anticoagulants, such as heparin or fondaparinux.

### 2.4. Sample Population and Enrolment Strategy

The Centers for Disease Control and Prevention (CDC) uses Epi Info™ software version 7.2 to do a priori statistical sample size calculations. The sample size was calculated by establishing a 99% confidence level, a 10% margin of error, and the fact that the population is finite. The study contained a final sample of 130 patients. During the study period, a consecutive sampling method was utilized to select the study participants. The study did not rely on a formal sample size calculation for agreement analysis. It utilized all consecutive prescriptions during the six-month study period that met the inclusion criteria. It should be noted that the sample size of 130 prescriptions indicates that it exhausted all the eligible cases in the study period. This method also applies to exploratory data analysis aimed at database agreement comparison.

### 2.5. Study Procedure

The study included prescription records of participants who met the eligibility requirements. Relevant demographic, clinical, and medication-related data were extracted electronically from patient health records and documented in a structured data collection form. Medication-related data included drug names, dosage, frequency, and duration of therapy, along with documented comorbid conditions. Only prescriptions with complete medication information were included in the analysis to ensure data adequacy and reliability.

Two subscription-based databases—Micromedex (MM) and Lexicomp (LC)—and one free online resource, Drugs.com (DC), were used. Micromedex (IBM Corporation) is a widely used clinical decision-support tool that provides evidence-based information on drug dosing, drug interactions, and adverse effects. LC (Wolters Kluwer Health) offers comprehensive drug monographs, interaction checkers, and clinical management recommendations. DC is a publicly accessible resource that consolidates information from multiple sources, including the FDA and other references, offering interaction checking for patients and clinicians. These platforms are commonly used in clinical practice, regularly updated, and include tools to classify the severity and clinical relevance of interactions (Supplementary Table S1).

All pDDIs concerning at least 1 anticoagulant were considered in the analysis, even if the pDDI was not related to the anticoagulant and it was a pDDI case between the administered drugs. All identified pDDIs were recorded in an Excel spreadsheet, sorted, and compared based on severity, evidence documentation, and recommended clinical management across the three resources. DC did not provide clear documentation or evidence-level classifications for the pDDIs.

Evaluating the clinical management techniques or advice given for the identified pDDIs in all three databases, different recommendations were found. Clinical recommendations by MM included adjusting the dose, changing the frequency of dosing, using the drug with caution, changing the therapy, not recommending it, taking no action, and monitoring it. DC, on the other hand, suggested changing the dosage interval, changing the dose (increasing or decreasing it), monitoring drug therapy, no change or action, and generally avoiding the drug or not recommending it. Clinical recommendations by LC were confined to avoiding use, adjusting the dose (increasing or decreasing it), monitoring, no change/action, and changing therapy. To enable a more meaningful and clear comparison and interpretation across the databases, the heterogeneous clinical management strategies were harmonized and consolidated into five pre-defined categories, as shown in Table 1.

#### 2.5.1. Scoring of Severity, Evidence, and Clinical Management

Similarly, a methodological framework was applied across the three databases by assigning a numerical score for each identified pDDI based on three dimensions, namely severity, quality of evidence, and clinical management. Severity ratings were coded as ‘1 = Minor,’ ‘2 = Moderate,’ ‘3 = Major,’ and ‘4 = Contraindicated.’ Evidence quality was coded as ‘1 = Excellent,’ ‘2 = Good,’ ‘3 = Fair,’ and ‘4 = Poor. Clinical management recommendations were coded as 1 = No action needed, 2 = Monitor only, 3 = Dose or regimen adjustment, 4 = Use with caution, and 5 = Avoid/Substitute. Where terminology differed slightly between databases, the original terms were mapped to the closest matching category according to these definitions to ensure consistency. This scoring framework, similar in structure to recommendations in the drug–drug interaction evidence assessment literature, ensured systematic comparison of severity levels, strength of evidence, and management advice across all three resources [15,16,17,18]. The framework was developed by a panel of three researchers with expertise in clinical pharmacy and pharmacology, each having prior experience in drug–drug interaction research, therapeutic drug monitoring, and evidence-based evaluation of prescribing practices. Their contribution, supported by a strong academic and clinical profile, to the development of a robust and practically useful scoring methodology was remarkable.

#### 2.5.2. Data Analysis

All the relevant data extracted from the databases were reviewed for completeness and consistency before inclusion. Only those records with complete medication details were included in the analysis. The data were then transferred to the Microsoft Excel spreadsheet, and the analysis was performed using IBM’s Statistical Package for the Social Sciences (SPSS) version 29.0 for Windows.

A structured analytical approach was adopted to evaluate inter-database variability and agreement. Descriptive statistics were used to summarize prescription-level and pDDI event-level data. Inferential statistical analyses were conducted using unique interaction pairs to assess differences and agreement across databases.

McNemar’s test was applied to evaluate the database consistency across severity, evidence, and management for each pDDI. Given the categorical nature of the data, McNemar’s test was applied to assess differences in paired categorical classifications between databases. Although the original interaction classifications were multi-categorical, the test was used to evaluate differences in paired proportions within specific categories. The results should therefore be interpreted with caution, as McNemar’s test is traditionally designed for binary outcomes.

The Wilcoxon signed-rank test was used to study overall differences between pairs of these ordinal variables, while the Friedman test was used to identify differences between all three databases. The study utilized post hoc Wilcoxon tests with Bonferroni adjustment (adjusted *p* < 0.017) to look at the important Friedman results.

Kendall’s coefficient of concordance (W) was calculated to evaluate the overall level of agreement among the databases in ranking pDDIs. Spearman correlation was used to look at the links between severity, evidence, and management scores. Fleiss’ Kappa (for numerous raters) and Cohen’s Kappa (for pairs) were used to see how much the databases agreed with each other. The study fixed the level of significance at *p* < 0.05.

For all inferential statistical analyses (including McNemar, Wilcoxon, Friedman, and agreement statistics), unique interaction pairs (n = 107) were used as the unit of analysis. Prescription-level and pDDI event-level data were used exclusively for descriptive purposes. This approach was adopted to avoid non-independence caused by repeated occurrences of the same interaction pair across multiple prescriptions. Where possible, the interactions were limited to all three databases to enhance consistency and avoid volume duplication.

## 3. Results

### 3.1. Socio-Demographic Characteristics of the Study Populations

In this study of 130 prescriptions, 58.46% were for women, and the majority were between the ages of 61 and 90 (63.07%). Almost half of the prescriptions were for patients from the UAE (45.38%). More than 90% of the patients included had one or more comorbidities, with the greatest (37.69%) having three to four. The most common comorbidities were cardiovascular and endocrine disorders. Neurological and psychiatric conditions were also identified, supporting the use of CNS-acting medications in a subset of patients. The most common reason for giving anticoagulant therapy was atrial fibrillation (66.15%), followed by deep vein thrombosis (20.76%). The average number of medications for each patient was 8.95 ± 4.73 (range: 2–18) per prescription (Table 2).

### 3.2. Identification of pDDIs from Different Resources

**Figure 1:**
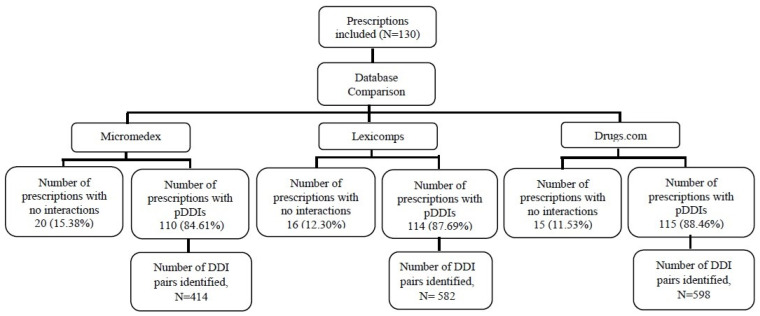
Schematic diagram showing the study sample size.

A total of 1164 drugs were identified, which yielded 3237 pDDIs involving 1143 unique drug pairs. DC reported 598 drug-interacting pairs, LC reported 582, and MM reported 414 drug-interacting pairs (Figure 1).

**Figure 2:**
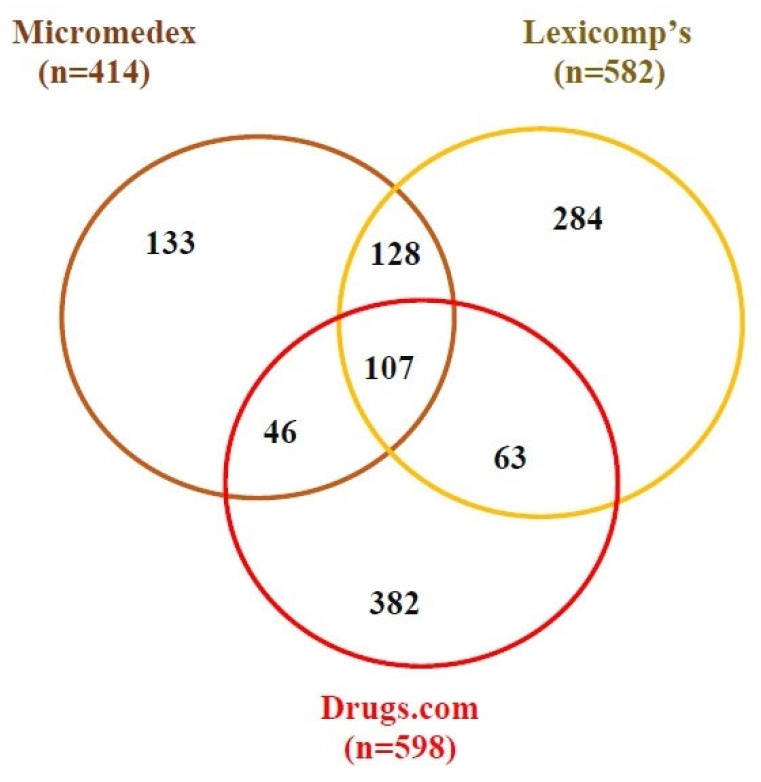
Overlap of potential drug–drug interactions among the three databases. In total, 1143 different pDDIs were identified from the patient cohort (n = 130). The Venn diagram shows the intersections of pDDIs between the databases. The circles and intersecting sets are drawn approximately to scale.

Of all medication interaction pairings, 799 (69.90%) were only found in one database. DC reported the most (382, 47.80%), followed by LC (284, 35.54%) and MM (133, 16.64%). Also, two of the three database resources reported 237 drug-interacting pairs (20.73%). MM and LC reported the most in common, with 128 (54.0%), followed by LC and DC, which reported 63 (26.58%). MM and DC had the lowest number of drug interactions (46, 19.30%). All three database resources reported the other 107 (9.36%) medication interactions (Figure 2).

### 3.3. Analyzing Severity, Evidence Quality, and Clinical Management Strategies Across Three Drug Interaction Databases

MM reported that 110 out of 130 (84.61%) prescriptions had pDDIs. They found 766 pDDIs and 414 medication-interacting pairings. The other 20 prescriptions (15.38%) did not have any drug–drug interactions. They were mostly (401, 52.34%) in the “moderate” category, followed by “major” (343, 44.77%) and “minor” (22, less than 3%). It was also noted that more than 462 (60%) of pDDIs were scored as having reasonable evidence, followed by over 35% (266) as having good evidence, and less than 5% (38) as having excellent evidence quality. Regarding management approaches, 508 (66.31%) of the pDDIs were to monitor therapy, 141 (18.40%) required dose adjustment, 66 (8.61%) recommended to use with caution, 30 (3.91%) recommended to avoid such combination, and the remaining 21 (2.74) pDDIs reported no action required.

LC reported 114 of the 130 prescriptions with pDDIs, 87.69% of the total. There were 1172 pDDIs and 582 medication-interacting pairings. There were 247 (21.1%) minor pDDIs, 865 (73.8%) moderate pDDIs, and 64 (5.5%) major pDDIs, based on the seriousness of the interaction. Considering the evidence quality, 867 (73.97%) were considered fair, 241 (20.56%) were considered good, 44 (3.75%) were considered excellent, and 20 (1.70%) were considered in the low category. Concerning the numerous management techniques, 832 (70.98%) called for close monitoring, 221 (18.85%) considered no action should be taken, 106 (9.04%) suggested changing therapy, and 13 (1.10%) recommended avoiding drug combinations.

DC reported that 115 (88.46%) out of 130 prescriptions had pDDIs. It also reported 1299 pDDIs and 598 drug interaction pairings. Of them, 1083 (83.37%) were classified as moderate, 114 (8.77%) were rated as significant, and 102 (7.85%) were rated as minor in severity. Considering the different management strategies, 1048 (80.67%) of the pDDIs were to monitor therapy, 96 (7.39%) were to avoid the drug combination, 80 (6.15%) were to use with caution, and the remaining 75 (5.77%) recommended adjusting the dose and/or the frequency interval.

### 3.4. Inter-Database Variability in Severity, Evidence Documentation, and Clinical Management Classification of pDDIs

There were 107 pDDIs found in all three DIDs, with varying classifications for each category. All databases agreed that 29 (27.10%) pDDIs were moderate and 10 (9.34%) pDDIs were major. But none of the three databases reported any of the pDDIs to be minor. As examples, MM reported the interaction between Amiodarone and Dabigatran as severe; however, LC and DC reported it as only moderate. Similarly, MM classified Apixaban and Diclofenac as major interactions, LC rated it as minor, and DC rated it as moderate. A few examples are presented in Table 3, and a detailed list is provided in Supplementary Table S2.

### 3.5. Pairwise Comparison of Severity, Evidence, and Management of pDDIs Across Three Databases

In terms of evidence level, 37 (34.57%) pDDIs were evaluated as fair by all databases, nine (8.41%) were rated as good, and only three (2.80%) pDDIs received exceptional evidence rating from all sources. All three databases suggested monitoring of 30 (28.03%) pDDIs as part of their management recommendations. There were only two (1.86%) pDDIs for which dose adjustment was recommended, and there were also only two (1.86%) for which to avoid/substitute medications were recommended. Interestingly, all three databases agreed that only one medication pair (0.93%) needed no intervention.

LC reported the most mild interactions (14, 13.08%), and DC reported the most moderate interactions (78, 72.89%), while MM reported the most major interactions (65, 60.74%). Using the McNemar test to compare LC with MM (*p* = 0.002) and DC (*p* = 0.013) for moderate drug interactions showed that there were statistically significant differences. There was also a similar difference when comparing MM to LC (*p* < 0.001) and DC (*p* < 0.001) for moderate and serious medication interactions. There was no statistical difference between the levels of evidence rating in LC and MM. There was also a statistically significant difference in management suggestions between MM and LC for some of the management categories (*p* < 0.001) and between DC and “monitor only” and “use with caution” (*p* < 0.001) (Table 4).

### 3.6. Overall Comparative Analysis of Severity, Evidence, and Management Across Databases

Using the Wilcoxon signed-rank test, there were differences in severity assessments between Micromedex (MM) and both Lexicomp (LC) (Z = −6.689, *p* < 0.001) and Drugs.com (DC) (Z = −5.591, *p* < 0.001), as well as between LC and DC (Z = −2.828, *p* = 0.005). Conversely, no differences were found in the evidence quality across the databases (Z = −0.800, *p* = 0.423). For classifications pertaining to clinical management, all pairwise comparisons involving MM yielded significant differences (*p* < 0.001).

The Friedman test further demonstrated a statistically significant difference in variation in severity ratings across all three databases (χ^2^(2) = 66.919, *p* < 0.001). For post hoc analysis, the Wilcoxon signed-rank test indicated that MM was significantly different from both LC (Z = 5.537, *p* < 0.001) and DC (Z = 3.999, *p* < 0.001), and the same differences were noted concerning management strategies for MM, LC (Z = 5.024, *p* < 0.001) and DC (Z = 3.999, *p* < 0.001) (Table 5).

Overall, these findings indicate substantial inter-database variability, particularly in severity and management classifications, with relatively consistent findings observed for evidence quality across databases.

Evaluation of the overall level of agreement among the three interaction databases using Kendall’s W indicated a moderate agreement for severity classification (χ^2^(2) = 66.919, W = 0.313) and a weak agreement for clinical management recommendations (χ^2^(2) = 54.351, W = 0.254).

### 3.7. Correlation Between Databases for Severity, Evidence Quality, and Management Ratings

There was a moderate positive and statistically significant association between MM and LC (ρ = 0.276, *p* = 0.004) and between MM and DC (ρ = 0.275, *p* = 0.004) for severity ratings. There was a greater link between LC and DC (ρ = 0.478, *p* < 0.001). MM had moderate positive correlations with DC for management classifications (ρ = 0.474, *p* < 0.001); however, there were no significant correlations between the databases for evidence quality levels (Table 6).

### 3.8. Agreement of pDDI Severity, Management, and Evidence Quality Classification Among Three Databases

Fleiss’ Kappa statistics showed that the three databases agreed on severity classification for minor interactions (κ = 0.21, *p* < 0.001), but not very much for moderate (κ = 0.14) and significant (κ = 0.17) categories. There was just a little consensus on how severe the cases were (κ = 0.16). There was only fair agreement for “No Action Needed” (κ = 0.23) and “Monitor Only” (κ = 0.23) in the management classification. There was only a little consensus among overall management (κ = 0.06) (Table 7).

## 4. Discussion

Anticoagulant medication and prescription for patients with cardiovascular illnesses is a common practice, and that makes patients susceptible to potential drug–drug (pDDI) interactions [9]. This work analyzed data consistency about potential drug–drug interactions (pDDIs) obtained from three popular drug databases: MM, LC, and DC. Polypharmacy is a practice that is prevalent among patients with significant comorbidities. Therefore, it creates a substantial concern regarding the risk of clinically significant drug–drug interactions (DDIs) within a practice. Thus, this illustrates the urgency to provide reliable, accurate, and current drug interaction database (DID) evaluations in the clinical context. It has been found that DIDs are inconsistent with respect to the nature of the info and the way they classify and describe pDDIs. DC documented the highest pDDIs among the three, despite LC having more pDDIs documented as known, as opposed to MC, which reported the least. In all the databases, it is observed that there is minimal agreement, while, in fact, the alignment is mostly noted between MM and LC. This highlights the overall contradictions that a clinician would experience when relying exclusively on one database for making clinical determinations. Additionally, only 9.36% of the overall pDDIs as reported in all three screening tools were noted. It is noteworthy that this finding is expected within previously published works, but it shows a low level of published findings within Germany (18.88%) and France (13.0%), while a relatively high level of the findings in China (4.88%) [19,20,21]. Some of these variations may correlate to variances in physicians’ prescribing practices, the databases selected, and the drugs and drug classifications included in the frameworks that are considered in the studies. There have been a great deal of discrepancies and variances between the clinical decision support systems (CDSS) and DIDs with respect to pDDIs, to the level of the clinical impact, and to the mechanisms of action and management of pDDIs [22,23,24].

Prior studies have found that inconsistencies between drug interaction databases can have direct implications for prescribing behavior, possibly leading to avoidable adverse drug interactions [20,23]. Reported concordance for DDI resources across other fields has been between 5% and 44%, emphasizing that variability is an issue across all settings [25,26,27,28]. This has been cited as a barrier for the adoption of solutions and has been found to decrease confidence in DDI tools [26]. Given these studies, severe inconsistencies were found across the DDI databases in all three major domains that were used in the research.

### 4.1. Severity Classification

There is cross-departmental heterogeneity in the classification of severity. This has been noted in databases, and the scarcity of agreement has been established. There is a discord among minor interactions, and there is a major and moderate discord where providence in the domain solutions tends to decrease the absence of labeling. This is more evident in the classification of these moderate and major interactions. Single sources should not be the only ones relied on for a classification norm. It has been noted that not only do databases differ in the classification, but also the drug resources that have been relied upon in the literature [19,21,29]. This gradation has also shown the differences that pertain to clinical safety that have to be compromised to still show that patient safety can be a larger issue [22].

Like previous studies, agreement across databases was less than fair. Despite statistical agreement, Fleiss’ kappa values showed a low scope of agreement. According to the criteria, the values corresponded to close-to-slight agreement, demonstrating a lack of significant collaboration between the databases. Hence, the clinical applicability of the findings was limited more by the scope of agreement than the *p*-values. For instance, studies on cardiovascular drugs showed substantial agreement, although not more than very poor or almost perfect. Research on older outpatient populations demonstrated few studies that were only moderately consistent [20,30,31]. These observations reinforce that severity classifications are particularly prone to variability across drug interaction databases. The lack of standardized definitions for “minor,” “moderate,” and “major” is a key factor underlying this variation and has been highlighted in the literature as a major limitation to cross-database comparability [25].

### 4.2. Documentation of Evidence

The classification of evidence across all databases could not be subject to comparative analysis because the online databases did not provide levels of evidence. Still, some evidence-rating agreement was noted between MM and LC, and some discrepancies were noted in defining and applying some evidence categories, such as “poor” or “fair” evidence. These discrepancies represent a significant obstacle to providing clinicians with quality evidence and may result in underestimating the clinical importance of the interactions. Previous studies reported similar evidence-rating discrepancies and noted that sources of drug information employed different evidence-rating systems [10,13,32]. These discrepancies represent a significant obstacle to providing clinicians with quality evidence and may result in underestimating the clinical importance of the interactions. Previous studies also reported similar evidence-rating discrepancies and noted that sources of drug information employed different systems of rating evidence [10,13,32]. Since the level of evidence rating and the quality of evidence are interrelated with clinicians’ level of certainty in their judgment, these discrepancies may result in clinicians either being overly reticent to prescribe or may result in clinicians not adequately perceiving safety concerns. Interestingly, evidence was rated as “Excellent” to high certainty, and evidence rated as “Fair” to low certainty was categorized in accordance with higher consistency in the literature. Another area of the literature was provided to describe that many resources providing details on drug interactions were not clear on the type and level of evidence that supports the recommendations [21,27].

### 4.3. Clinical Management of pDDIs

There was evidence of more discrepancies across clinical management recommendations than there were across evidence or severity classifications. Such discrepancies, which provide little information, may lead to clinical significance, as management recommendations of “avoid,” “monitor,” or “no action” differ in a way that would lead more to treatment discrepancies and less to safety concerns of evidence of treatment or management of drug interactions. The studies in the UK and Norway that analyzed management recommendations also noted discrepancies within drug information databases, which supports our analysis [10,33].

The existing data indicates that variances can be attributed to changes in update cycles, evaluation methods, and the drug classes under analysis [29,34]. We examined how clinicians evaluate the management recommendations across multiple databases, not just one. The lack of alignment in recommendations can lead to high alert fatigue, and that is concerning. No less than 90–95% of the DDI alerts in CDSS were typically ignored due to a lack of specificity or alignment in the recommendations [34,35]. Lastly, our research also showed that many DDI tools did not provide clear and actionable recommendations, a limitation noted in our research [36]. Lastly, some analyses revealed the moderation of some databases. It shows that moderation was achieved, although a lack of alignment was documented at the same time. It indicates a considerable lack of alignment and a variance in databases, and the interaction data presented should be interpreted carefully by the clinicians.

### 4.4. Recommendations/Practical Implementations

To make DIDs clinically valid, relevant, and reliable, first, a clinically relevant and evidence-based definition for pDDIs and a universal framework for classifying severity and evidence quality should be implemented. To this end, the WHO-UMC classification model or the GRADE model should be used, as they both present a balanced evidence-based approach to high-quality, peer-reviewed evidence within the context of the study, the intervention, and the population. Establishing coherent and systematic rules concerning evidence collection, similar to those applied to Cochrane reviews and clinical practice guidelines, will ensure that evidence of interactions is systematically and rigorously incorporated. To enhance clinical relevance, prior studies suggest the implementation of tiered alerting systems in CDSS. In these systems, high-severity interactions alert the clinician, thereby managing alert fatigue and improving clinician promptness, while low-severity interactions are presented in a less intrusive manner [37,38,39]. DDI alerts can become more useful and relevant for the clinician by incorporating specific patient-related risk elements such as age, presence of renal or hepatic abnormalities, presence of other co-morbidities, and the presence of genetic polymorphisms such as CYP2C9 or VKORC1 [40]. These also allow the DIDs to be more relevant as individualized patient alerts. The addition of particle alerts and patient-centered interaction risk scoring will make CDSS more user-friendly by incorporating tiered alerting systems. AI-based CDS systems that improve in real time are also useful.

This approach cuts down alert fatigue and provides appropriate and precise responses. Regulatory bodies and authorities, such as the FDA, EMA, or WHO, could cooperate to design and create a gold standard for drug–drug interaction references. These standards would provide a reliable benchmark for the harmonization of drug interaction databases. There is a need for future multicenter studies to assess which classification systems best predict patient risks by linking real-world clinical outcomes. For international harmonization and targeted education, the commitment to using drug interaction tools in a safe and effective manner will strengthen efforts the most.

### 4.5. Strengths and Limitations

An attempt to assess the consistency of real-world patient data across the three databases in this study increased the relevance, scientific value, and real-world in situ applicability of the results to clinical practice. This study examined three databases and analyzed pDDIs in terms of severity and clinical evidence, and provided clinical management of pDDIs. This research is distinguished from other studies by leveraging a standardized, ingredient-centric model to evaluate drug interaction management across different sources. Prior studies relied on one or two drug interaction databases and were singular in focus on drug interaction severity, clinical evidence, or management due to a lack of an integrative comparison of severity, evidence quality, and management recommendations. In addition, the use of inter-rater reliability statistics provided a formal and demonstrable basis for the agreement to strengthen the comparison.

There were several limitations to this study. First, only data available at the time of retrieval were considered; consequently, the results reflected the state of the resources at that specific period. Although major updates to these databases were infrequent, subsequent changes may not have been captured in this analysis. Second, the study population was limited to a single center, which may have reduced the generalizability of the findings. Third, DC did not provide evidence levels, preventing a complete triadic comparison in this aspect. Fourth, the study did not assess the clinical relevance of the identified pDDIs against actual adverse event outcomes, and therefore, it only evaluated theoretical clinical risk. Certain drug interactions depended on the route of administration or dose. Although systemic drugs were included, differences between intravenous and oral routes or dose-dependent interactions were not analyzed. There is a direct relationship between the agreement concordance values and the harmonization process, so any oversimplification or lack of equivalence in the mapped categories could have affected the level of concordance that was described. Although the study design utilized McNemar’s test, which is typically applied to binary outcomes, the test was used in the context of categorical data, and therefore, results must be interpreted with caution. The agreement concordance values are directly dependent on the harmonization framework applied across databases. Therefore, any oversimplification or lack of clinical equivalence in the mapping of categories may introduce misclassification bias and substantially influence the reported agreement metrics. In particular, categories such as “monitor closely,” “therapy modification,” “avoid use,” and “use with caution” may not necessarily reflect identical clinical thresholds across different databases. This semantic non-equivalence represents an important methodological limitation and should be considered when interpreting the agreement results. Information on patient educational level was not available in the electronic health records, which may limit the assessment of its potential impact on medication use and drug–drug interactions.

## 5. Conclusions

Inconsistencies in pDDI classification were observed among DIDs and across three key dimensions of the resources. Clinicians may experience challenges in clinical decision-making due to heterogeneity in various dimensions, including evidence quality, severity, and management guidance. The incorporation of advanced clinical decision support systems into electronic health records may improve the credibility and clinical value of drug interaction resources, especially if combined with high-quality drug interaction evidence and developed criteria. While pDDIs provide some information and guidelines, prescribers are encouraged to be proactive, use multiple reference tools instead of a single tool, and incorporate standardized, evidence-based frameworks into clinical decision support strategies to improve consistency, reliability, and, ultimately, patient outcomes.

## Figures and Tables

**Table 1:** Management classification of drug interactions.

Recommendation/ Management	Description	Included Terms
No action needed	No clinical concern or change required. No dose, regimen, or therapy adjustment is necessary.	No action, no change, no dose adjustment.
Monitor only	Monitor the patient or therapy (e.g., labs, side effects), but no changes to the drug, dose, or regimen.	Monitor, monitor closely, monitor/low dose, use with caution/monitor.
Dose or regimen adjustment	Modify how the drug is given—such as dose amount, dosing interval, or frequency—without changing the drug.	Adjust dose, dose adjust, decrease dose, dose reduction, frequency adjust, modify dosing frequency, monitor/dose adjust, monitor/dose frequency.
Use with caution	Use is not contraindicated, but requires caution. May or may not include monitoring, based on clinical judgment.	Use with caution, caution.
Avoid/substitute	Avoid the drug combination or substitute with a safer alternative. Indicates a potentially serious or clinically significant interaction.	Avoid, not recommended, alternative, therapy modification.

**Table 2:** Baseline socio-demographic profile of the study populations (n = 130).

Clinical Characteristics	Frequency (n = 130)	Percentage
Gender		
Female	76	58.46
Male	54	41.53
Age (in Years)		
≤30	4	3.07
31–60	42	32.30
61–90	82	63.07
>90	2	1.53
Age (years) (Mean ± SD)	64.9 ± 17.2	
Nationality		
Asians	26	20
United Arab Emirates (UAE)	59	45.38
Middle Eastern countries other than the UAE	37	28.46
Others	8	6.15
Comorbidities		
Nil	8	6.15
1–2	41	31.53
3–4	49	37.69
≥5	32	24.61
Types of Comorbidities		
Endocrine disorders	77	18.37
Cardiovascular diseases	191	45.58
Renal disease	24	5.72
Respiratory diseases	12	2.86
Hematological	18	4.29
Gastrointestinal	11	2.62
Musculoskeletal disorders	22	5.25
Neurological	11	2.62
Psychiatric	12	2.86
Dermatological	6	1.43
Ophthalmology	9	2.14
Genitourinary system	8	1.90
Infectious diseases	4	0.95
Others	14	3.34
Current diagnosis		
Atrial fibrillation	86	66.15
Atrial flutter	5	3.84
Deep vein thrombosis	27	20.76
Pulmonary embolism	11	8.46
Mitral valve regurgitation	1	0.76
Number of drugs per prescription		
1–5	26	20.0
6–10	49	37.69
11–15	40	30.76
≥16	15	11.53
Total number of medications	1164	
Average number of medications per patient (Mean ± SD)	8.95 ± 4.73	

SD: Standard deviation.

**Table 3:** Selected examples of severity, management, and evidence classifications of pDDIs across three databases (n = 107).

Drug Interaction Pairs	MM			LC’s			DC	
	Severity	Management	Evidence	Severity	Management	Evidence	Severity	Management
Amiodarone–Clopidogrel	Major	Avoid	Fair	Minor	No action	Fair	Moderate	Monitor
Apixaban–Clopidogrel	Major	Monitor	Fair	Major	Monitor	Good	Major	Monitor
Aspirin–Calcium carbonate	Moderate	Adjust dose	Fair	Minor	No action	excel	Moderate	Monitor
Digoxin–Amiodarone	Major	Dose red	Excellent	Major	Therapy modification	excel	Major	Dose reduction
Amiodarone–Rivaroxaban	Moderate	Monitor	Good	Moderate	Monitor	Good	Moderate	Monitor
Atorvastatin–Dabigatran	Major	Avoid	Good	Minor	No action	Good	Minor	No action
Apixaban–Diltiazem HCL	Major	Use with caution	Good	Major	Monitor	Fair	Moderate	Monitor
Apixaban–INH/PZN/RIF	Major	Avoid	Excellent	Major	Avoid use	Good	Major	Monitor
Bisoprolol–Meloxicam	Moderate	Monitor	Good	Moderate	Monitor	Fair	Moderate	Monitor
Apixaban–Celecoxib	Major	Monitor	Fair	Moderate	Monitor	Good	Major	Monitor
Furosemide–Insulin lispro	Moderate	Monitor/dose adjustment	Fair	Moderate	Monitor	Fair	Moderate	Monitor
Carvedilol–Empagliflozin	Moderate	Dose adjustment	Good	Moderate	Monitor	Fair	Moderate	Monitor
Levothyroxine–Pantoprazole	Moderate	Monitor	Good	Minor	No action	Fair	Moderate	Monitor
Carbamazepine–Levothyroxine	Moderate	Monitor	Fair	Moderate	Monitor	Good	Moderate	Monitor
Furosemide–Metformin	Moderate	Monitor	Fair	Moderate	Monitor	Fair	Moderate	Monitor
Aspirin–Rivaroxaban	Major	Use caution/Monitor	Fair	Major	Monitor	Good	Major	Monitor
Carbamazepine–Rivaroxaban	Major	Avoid	Good	Major	Avoid use	Good	Major	Avoid
Apixaban–Aspirin	Major	Monitor	Good	Major	Monitor	Good	Major	Monitor
Atenolol–Empagliflozin	Moderate	Monitor	Good	Moderate	Monitor	Fair	Moderate	Monitor
Atorvastatin–Clopidogrel	Moderate	Therapy modification	Excellent	Minor	No action	Good	Moderate	Monitor
Bisoprolol–Empagliflozin	Moderate	Dose adjustment	Good	Moderate	Monitor	Fair	Moderate	Monitor
Cabergoline–Quetiapine	Moderate	Avoid	Fair	Moderate	Avoid use	Fair	Major	Avoid
Carbamazepine–Levetiracetam	Moderate	Use caution/Monitor	Good	Moderate	Monitor	Fair	Moderate	Monitor
Carvedilol–Glimepiride	Moderate	Dose adjustment	Good	Moderate	Monitor	Fair	Moderate	Monitor
Celecoxib–Rivaroxaban	Major	Monitor	Fair	Moderate	Monitor	Good	Major	Monitor closely
Clopidogrel–Dexlansoprazole	Moderate	No action	Excellent	minor	No action	Good	Minor	No change
Diclofenac–Rivaroxaban	Major	Monitor	Fair	Minor	Monitor	Fair	Moderate	Monitor
Apixaban–Amiodarone	Major	Monitor	Good	Moderate	No action	Fair	Moderate	No dosage adjustment
Furosemide–Hydralazine	Minor	Monitor/dose adjust	Good	Moderate	Monitor	Fair	Minor	No action
Atenolol–Calcium carbonate	Minor	Avoid	Good	Minor	No action	Fair	Moderate	Adjust dosing interval
Calcium carbonate–Ferrous fumarate	Minor	Avoid	Fair	Minor	Therapy modification	Fair	Moderate	adjust dose interval
Metformin–Perindopril	Moderate	Monitor	Fair	Moderate	No action	poor	Moderate	Monitor
Spironolactone–Valsartan	Moderate	Monitor	Fair	Major	Monitor	excel	Major	Monitor closely

**Table 4:** Pairwise comparison between the three databases regarding severity, evidence classification, and management of pDDIs (McNemar Test).

	MM N (%)	LC’s N (%)	DC N (%)	P (MM vs. LC’s)	P (MM vs. DC)	P (LC’s vs. DC)
Severity level						
Mild	03 (2.80)	14 (13.08)	05 (4.67)	0.002 *	0.687	0.013 *
Moderate	39 (36.44)	76 (71.02)	78 (72.89)	<0.001 *	<0.001 *	0.728
Major	65 (60.74)	17 (15.88)	24 (22.42)	<0.001 *	<0.001 *	0.167
Evidence classification						
Excellent	08 (7.47)	09 (8.41)	--	1.000		
Fair	61 (57.0)	68 (63.55)	--	0.418		
Good	38 (35.51)	28 (26.16)	--	0.203		
Poor	--	02 (1.86)	--	--		
Management						
No action needed	01 (0.93)	13 (12.14)	04 (3.73)	<0.001	0.250	0.012
Monitor only	40 (37.38)	74 (69.15)	71 (66.35)	<0.001	<0.001 *	0.556 *
Dose or regimen adjustment	23 (21.49)	04 (3.73)	18 (16.82)	<0.001	0.458	<0.001
Use with caution	18 (16.82)	00 (0.0)	00 (0.0)	<0.001	<0.001 *	--
Avoid/substitute	25 (23.36)	16 (14.95)	14 (13.08)	0.152	0.031 *	0.804

* *p* < 0.05 indicates statistical significance. MM—Micromedex; LC—Lexicomp’s; DC—Drugs.com.

**Table 5:** Distribution and pairwise comparison of severity and management of pDDIs across three databases.

Comparison Type	Test Used	Database Pair	Test Statistic	*p*-Value	Adjusted *p*-Value
Severity	Friedman Test	All three	χ^2^(2) = 66.919	<0.001 *	—
	Wilcoxon Signed-Rank (Post hoc for Friedman)	LC vs. DC	Z = −1.538	0.124	0.372
		LC vs. MM	Z = 5.537	<0.001 *	<0.001 *
		MM vs. DC	Z = 3.999	<0.001 *	<0.001 *
Management	Friedman Test	All three	χ^2^(2) = 54.351	<0.001 *	—
	Wilcoxon Signed-Rank (Post hoc for Friedman)	LC vs. DC	Z = −1.025	0.305	0.916
		LC vs. MM	Z = 5.024	<0.001 *	<0.001 *
		MM vs. DC	Z = 3.999	<0.001 *	<0.001 *

* *p* < 0.05 indicates statistical significance.

**Table 6:** Analysis of severity, evidence, and management ratings between databases by using Spearman’s correlation of (n = 107).

Comparison	Category	Spearman’s ρ	*p*-Value	Interpretation
MM vs. LC’s	Severity	0.276	0.004 *	Moderate positive, significant
MM vs. DC		0.275	0.004 *	Moderate positive, significant
LC’s vs. DC		0.478	0.000 *	Moderate positive, significant
MM vs. LC’s	Evidence	0.056	0.565	Very weak correlation, not significant
MM vs. LC’s	Management	0.206	0.033 *	Weak positive, significant
MM vs. DC		0.474	0.000 *	Moderate positive, significant
LC’s vs. DC		0.584	0.000 *	Strong positive, significant

* *p* < 0.05 indicates statistical significance; MM—Micromedex; LC—Lexicomp’s; DC—Drugs.com.

**Table 7:** Inter-database agreement on severity and management classification of potential drug–drug interactions across different databases by using Fleiss’ kappa.

**Severity**	**MM**	**LC**	**DC**	**Fleiss’ Kappa** **(95% CI)**	***p* Value**	**Strength**
	Minor	Minor	Minor	0.21 (0.09–0.32)	<0.001	Fair
	Moderate	Moderate	Moderate	0.14 (0.04–0.25)	0.01	Slight
	Major	Major	Major	0.17 (0.06–0.28)	0.002	Slight
	Overall			0.16 (0.07–0.25)	0.001	Slight
**Management**	No Action Needed	No Action Needed	No Action Needed	0.23 (0.13–0.34)	<0.001	Fair
	Monitor Only	Monitor Only	Monitor Only	0.23 (0.12–0.34)	<0.001	Fair
	Dose or Regimen Adjustment	Dose or Regimen Adjustment	Dose or Regimen Adjustment	0.15 (0.04–0.26)	0.008	Slight
	Use with Caution	Use with Caution	Use with Caution	−0.12 (−0.15–0.09)	1.00	Poor
	Avoid/Substitute	Avoid/Substitute	Avoid/Substitute	0.01 (−0.03–0.04)	0.722	Slight
	Overall			0.06 (0.02–0.09)	<0.001	Slight

Cohen’s Kappa indicated fair agreement (κ = 0.297, *p* = 0.002) for the “Excellent” evidence category between MM and LC. However, there was not much agreement for “Good” (κ = −0.084), “Fair” (κ = −0.069), and overall evidence classifications (κ = −0.024, *p* = 0.752).

## Data Availability

The data supporting the findings of this study are available from the corresponding author upon reasonable request.

## Supplementary Materials

The following supporting information can be downloaded at: https://www.mdpi.com/article/10.3390/medicina62050872/s1, Table S1: Severity, Documentation, and Management of pDDIs across Selected Drug Interaction Databases; Table S2: Comparison of severity, management and evidence classifications of pDDIs across three databases (n = 107).

## Abbreviations

The following abbreviations are used in this manuscript:

pDDIs	Potential drug–drug interactions
DOAG	Direct oral anticoagulants
UAE	United Arab Emirates
LC	Lexicomp
MM	Micromedex
DC	Drugs.com
DIDs	Drug interaction databases
