# Supplementary material for: Screening for Potential Drug–Drug Interactions in Patients Receiving Anticoagulant Therapy: A Comparison of Three Drug Interaction Databases for Consistency in Severity Rating, Evidence Classification, and Clinical Management
Source: Medicina (Kaunas). 2026 May 2;62(5):872. doi: 10.3390/medicina62050872 (PMC13208237; doi:10.3390/medicina62050872)
Supplement: Supplementary file 1 [file medicina-62-00872-s001.zip › medicina-4249547-supplementary.pdf]

Table S1 Severity, Documentation, and Management of pDDIs across Selected Drug Interaction Databases

	Micromedex	Lexicomp's	Drugs.com
**Severity**	Minor: The interaction would have limited clinical effects. Manifestations may include an increase in the frequency or severity of side effects but generally would not require a major alteration in therapy.<br>Moderate: The interaction may result in exacerbation of patient's condition and/or require an alteration in therapy Major: The interaction may be life- threatening and/or require medical intervention to minimize or prevent serious adverse effects Contraindicated - The drugs are contraindicated for current use<br>Unknown - Unknown	Minor: The effects are usually bearable and don't need medical help. Most of the time, therapy doesn't need to change.<br>Moderate: Effects may require medical help, such as changing the dose, monitoring, or getting additional care, but they are not life-threatening.<br>Major: The effects could be life-threatening, create lasting damage, fail to work as a treatment, or need hospitalization.	Minor: Minimally clinically significant. Minimize risk; assess risk and consider an alternative drug, take steps to circumvent the interaction risk and/or institute a monitoring plan.<br>Moderate: Moderately clinically significant. Usually avoid combinations; use it only under special circumstances.<br>Major: Highly clinically significant. Avoid combinations; the risk of the interaction outweighs the benefit.<br>Unknown - No interaction information available.
**Level of documentation**	Excellent - Controlled studies have clearly established the existence of the interaction<br>Fair - Available documentation is poor, but pharmacologic considerations lead clinicians to suspect the interactions exists (or) documentation is good for a pharmacologically similar drug.<br>Good Documentation strongly suggests the interaction exists, but well controlled studies are lacking<br>Unknown - Unknown	Excellent: Comprehensive, well-organized, high-quality evidence, clear recommendations, regularly updated.<br>Good: Complete and reliable evidence, mostly clear, generally up-to-date.<br>Fair: Adequate but limited evidence or clarity, may not be comprehensive.<br>Poor: Incomplete, minimal or outdated evidence, less reliable for decision-making.	N/A
**Management**	Recommendations included clinical monitoring, avoid combination, use with caution, dose/frequency adjustment, therapy modification, no action	Recommendations included avoid use, dose adjust (increase/decrease), monitor, no action, therapy modification	Management included dose adjust (increase/decrease), monitor closely, dose frequency adjusts, no action or no change, not recommended

N/A – Not Avaialable

Table S2 Comparison of severity, management and evidence classifications of pDDIs across three databases (n=107)

Drug interaction pairs	MM			LC's			DC	
	Severity	Management	Evidence	Severity	Management	Evidence	Severity	Management
Amiodarone - Clopidogrel	Major	Avoid	Fair	Minor	No action	Fair	Moderate	Monitor
Apixaban – Clopidogrel	Major	Monitor	Fair	Major	Monitor	Good	Major	Monitor
Apixaban – Escitalopram	Major	Monitor	Fair	Moderate	Monitor	Good	Moderate	Monitor
Aspirin - Calcium carbonate	Moderate	Adjust dose	Fair	Minor	No action	excel	Moderate	Monitor
Digoxin - Amiodarone	Major	Dose red	Excellent	Major	Therapy modification	excel	Major	Dose reduction
Amiodarone - Formoterol	Major	Avoid	Good	Moderate	Monitor	Fair	Moderate	Monitor
Bisoprolol - Formoterol	Major	Therapy modification	Fair	Moderate	Monitor	Good	Moderate	Avoid
Donepezil - Quetiapine	Major	Avoid	Fair	Moderate	Monitor	Fair	Moderate	Avoid
Duloxetine - Rivaroxaban	Major	Monitor	Fair	Moderate	Monitor	Good	Moderate	Monitor
Furosemide - Insulin degludec	Major	Monitor/dose adjust	Fair	Moderate	Monitor	Fair	Moderate	Monitor
Glibenclamide (INT) – Metformin	Major	Dose adjust	Fair	Moderate	Monitor	Fair	Moderate	Monitor
Hydrochlorothiazide - Metformin	Moderate	Monitor	Fair	Moderate	Monitor	Fair	Moderate	Monitor
Hydroxychloroquine - Sertraline	Major	Avoid	Fair	Moderate	Monitor	Fair	Major	Avoid
Bisoprolol - Glimepiride	Moderate	Dose adjustment	Good	Moderate	Monitor	Fair	Moderate	Monitor
Bisoprolol - Dapagliflozin	Moderate	Dose adjustment	Good	Moderate	Monitor	Fair	Moderate	Monitor
Amiodarone - Rivaroxaban	Moderate	Monitor	Good	Moderate	Monitor	Good	Moderate	Monitor
Amiodarone - Rosuvastatin	Moderate	caution	Good	Major	No action	Fair	Moderate	Monitor
Atorvastatin - Dabigatran	Major	Avoid	Good	Minor	No action	Good	Minor	No action
Bisoprolol - Budesonide	Major	Monitor	Fair	Moderate	Monitor	Good	Moderate	Dosage adjustment
Apixaban – Ibuprofen	Major	Monitor	Fair	Moderate	Monitor	excel	Major	Monitor
Apixaban – Meloxicam	Major	Monitor	Fair	Moderate	Monitor	excel	Major	Monitor
Aspirin - Clopidogrel	Major	Monitor	Fair	Moderate	Monitor	Fair	Moderate	Monitor
Aspirin - Insulin degludec	Moderate	Use with caution/Monitor	Fair	Moderate	Monitor	Fair	Moderate	Monitor

Bisoprolol - Patiromer	Moderate	Frequency adjustment	Fair	Moderate	Therapy modification	Fair	Moderate	Adjust dose interval
Atenolol - Lacosamide	Major	Use caution/Monitor	Fair	Moderate	Monitor	Good	Moderate	Monitor
Apixaban – Diltiazem HCL	Major	Use with caution	Good	Major	Monitor	Fair	Moderate	Monitor
Apixaban - INH/ PZN/ RIF	Major	Avoid	Excellent	Major	Avoid use	Good	Major	Monitor
Bisoprolol - Meloxicam	Moderate	Monitor	Good	Moderate	Monitor	Fair	Moderate	Monitor
Apixaban – Levetiracetam	Major	Avoid	Fair	Moderate	Monitor	Fair	Moderate	Dose adjustment
Furosemide - Metolazone	Major	Use caution/Monitor	Good	Moderate	Monitor	Fair	Moderate	Monitor
Furosemide - Sucralfate	Moderate	Monitor/dose frequency	Fair	Moderate	Therapy modification	Fair	Moderate	Adjust dosing interval
Amiodarone - Glyburide	Moderate	Dose reduction	Fair	Moderate	Monitor	Fair	Major	Dose adjust
Amiodarone – Rifampin	Major	Dose reduction	Good	Moderate	Dose adjustment	Good	Major	Dose adjust
Amiodarone - Risperidone	Major	Avoid	Fair	Major	Therapy modification	Fair	Major	Avoid
Carvedilol - Repaglinide	Moderate	Dose adjustment	Good	Moderate	Monitor	Fair	Moderate	Monitor
Aspirin - Insulin glargine	Moderate	Use with caution/Monitor	Fair	Moderate	Monitor	Fair	Moderate	Monitor
Bisoprolol - Insulin degludec	Moderate	Dose adjustment	Good	Moderate	Monitor	Fair	Moderate	Monitor
Aspirin - Insulin lispro	Moderate	Use with caution/Monitor	Fair	Moderate	Monitor	Fair	Moderate	Monitor
Digoxin - Spironolactone	Major	Modify dosing frequency	Good	Moderate	Monitor	Fair	Minor	Monitor
Furosemide - Glyburide	Major	Dose adjustment	Fair	Moderate	Monitor	Fair	Moderate	Monitor
Apixaban – Ticagrelor	Major	Monitor	Fair	Major	Monitor	Good	Major	Monitor
Apixaban – Celecoxib	Major	Monitor	Fair	Moderate	Monitor	Good	Major	Monitor
Carvedilol - Insulin degludec	Moderate	Dose adjustment	Good	Moderate	Monitor	Fair	Moderate	Monitor
Carvedilol - Insulin lispro	Moderate	Dose adjustment	Good	Moderate	Monitor	Fair	Moderate	Monitor
Clopidogrel - Repaglinide	Major	Use caution/Monitor	Good	Major	Therapy modification	Good	Major	Avoid
Bisoprolol - Insulin lispro	Moderate	Dose adjustment	Good	Moderate	Monitor	Fair	Moderate	Monitor
Clopidogrel - Rivaroxaban	Major	Avoid	Good	Major	Monitor	Good	Major	Monitor closely

Dapagliflozin – Degludec Insulin	Major	Decrease dose	Fair	Moderate	Therapy modification	Fair	Moderate	Adjust dose
Indapamide - Metformin	Moderate	Monitor	Fair	Moderate	Monitor	Fair	Moderate	Monitor
Aspirin - Sertraline	Major	Use caution/Monitor	Excellent	Moderate	Monitor	Fair	Moderate	Monitor
Indapamide - Perindopril	Moderate	Dose reduction	Fair	Moderate	Monitor	Fair	Moderate	Monitor
Carbamazepine - Topiramate	Major	Avoid	Excellent	Major	Monitor	excel	Moderate	adjust dose
Carvedilol - Dapagliflozin	Moderate	Dose adjustment	Good	Moderate	Monitor	Fair	Moderate	Monitor
Aspirin - Spironolactone	Major	Monitor	Good	Minor	Dose increase	Fair	Minor	Dose increase
Furosemide - Insulin lispro	Moderate	Monitor/dose adjustment	Fair	Moderate	Monitor	Fair	Moderate	Monitor
Furosemide - Linagliptin	Major	Monitor/dose adjustment	Fair	Moderate	Monitor	Fair	Moderate	Monitor
Aspirin - Ticagrelor	Major	therapy modification	Fair	Major	Therapy modification	Fair	Moderate	Adjust dose
Carvedilol - Empagliflozin	Moderate	Dose adjustment	Good	Moderate	Monitor	Fair	Moderate	Monitor
Levothyroxine - Pantoprazole	Moderate	Monitor	Good	Minor	no action	Fair	Moderate	Monitor
Lisinopril - Valsartan	Major	Avoid	excel	Moderate	Therapy modification	excel	Major	Avoid
Carbamazepine - Levothyroxine	Moderate	Monitor	Fair	Moderate	Monitor	Good	Moderate	Monitor
Furosemide - Metformin	Moderate	Monitor	Fair	Moderate	Monitor	Fair	Moderate	Monitor
Aspirin - Meloxicam	Major	Not recommended	Fair	Major	Therapy modification	Good	Moderate	Not recommended
Aspirin - Perindopril	Major	Use caution/Monitor	Fair	Moderate	Monitor	Fair	Moderate	Monitor
Aspirin - Rivaroxaban	Major	Use caution/Monitor	Fair	Major	Monitor	Good	Major	Monitor
Carbamazepine - Rivaroxaban	Major	Avoid	Good	Major	Avoid use	Good	Major	Avoid
Acetaminophen - Carbamazepine	Major	Dose reduction	Good	Moderate	Monitor	Good	Moderate	Avoid
Amantadine - Orphenadrine	Major	Dose reduction	Fair	Moderate	Dose reduction	Fair	Moderate	Dose reduction
Apixaban – Aspirin	Major	Monitor	Good	Major	Monitor	Good	Major	Monitor

Amiodarone - Amitriptyline	Major	Avoid	Fair	Moderate	Monitor	Fair	Major	Avoid
Aspirin - Celecoxib	Major	Monitor	Fair	Moderate	Therapy modification	Good	Moderate	Not recommended
Quetiapine - Amantadine	Major	Caution	Fair	Moderate	Avoid use	Fair	Moderate	Dose reduction
Atenolol - Empagliflozin	Moderate	Monitor	Good	Moderate	Monitor	Fair	Moderate	Monitor
Atorvastatin - Clopidogrel	Moderate	Therapy modification	Excellent	Minor	No action	Good	Moderate	Monitor
Bisoprolol - Empagliflozin	Moderate	Dose adjustment	Good	Moderate	Monitor	Fair	Moderate	Monitor
Cabergoline - Quetiapine	Moderate	Avoid	Fair	Moderate	Avoid use	Fair	Major	Avoid
Budesonide - Amiodarone	Major	Avoid	Good	Moderate	Monitor	Fair	Moderate	Dose frequency
Carvedilol - Amiodarone	Major	Monitor	Fair	Moderate	Monitor	Good	Moderate	Monitor
Carbamazepine - Levetiracetam	Moderate	Use caution/Monitor	Good	Moderate	Monitor	Fair	Moderate	Monitor
Carvedilol - Glimepiride	Moderate	Dose adjustment	Good	Moderate	Monitor	Fair	Moderate	Monitor
Celecoxib - Rivaroxaban	Major	Monitor	Fair	Moderate	Monitor	Good	Major	Monitor closely
Clonazepam - Gabapentin	Major	Monitor/low dose	Fair	Moderate	Monitor	Good	Moderate	Monitor
Clopidogrel - Dexlansoprazole	Moderate	No action	Excellent	minor	No action	Good	Minor	No change
Diclofenac - Rivaroxaban	Major	Monitor	Fair	Minor	Monitor	Fair	Moderate	Monitor
Apixaban – Amiodarone	Major	Monitor	Good	Moderate	No action	Fair	Moderate	No dosage adjustment
Atorvastatin - Amiodarone	Moderate	Use alternative	Good	Moderate	Dose reduction	Fair	Moderate	Dose reduction
Furosemide - Hydralazine	Minor	Monitor/dose adjust	Good	Moderate	Monitor	Fair	Minor	No action
Mirtazapine - Quetiapine	Major	Use caution/Monitor	Fair	Moderate	Monitor	Good	Moderate	Avoid
Naproxen - Rivaroxaban	Major	Monitor	Fair	Moderate	Monitor	Excel	Major	Monitor closely
Amiodarone - Dabigatran	Major	Avoid	Good	Moderate	Monitor	Good	Moderate	Monitor
Apixaban – Diclofenac	Major	Monitor	Fair	Minor	Monitor	Fair	Moderate	Monitor
Aspirin - Lisinopril	Major	Use with caution/Monitor	Fair	Moderate	Monitor	Fair	Moderate	Monitor
Atenolol - Calcium carbonate	Minor	Avoid	Good	Minor	No action	Fair	Moderate	Adjust dosing interval
Bisoprolol - Celecoxib	Moderate	Monitor	Good	Moderate	Monitor	Fair	Moderate	Monitor

Calcium carbonate - Ferrous fumarate	Minor	Avoid	Fair	Minor	Therapy modification	Fair	Moderate	adjust dose interval
Donepezil - Escitalopram	Major	Use caution/Monitor	Good	Minor	No action	Fair	Moderate	Monitor
Ferrous sulphate - Pantoprazole	Moderate	Monitor	Fair	Minor	No action	Fair	Moderate	Monitor
Furosemide - Insulin glargine	Major	Monitor/dose adjust	Fair	Moderate	Monitor	Fair	Moderate	Monitor
Hydroxychloroquine - Quetiapine	Major	Avoid	Fair	Moderate	Monitor	Fair	Major	Avoid
Indapamide - Liraglutide	Major	Monitor/dose adjust	Fair	Moderate	Monitor	Fair	Moderate	Monitor
Levothyroxine - Omeprazole	Moderate	Monitor	Good	Minor	No action	Fair	Moderate	Monitor
Metformin - Perindopril	Moderate	Monitor	Fair	Moderate	No action	poor	Moderate	Monitor
Orphenadrine - Quetiapine	Major	Use caution/Monitor	Fair	Major	Avoid use	Fair	Moderate	Monitor
Spironolactone - Valsartan	Moderate	Monitor	Fair	Major	Monitor	excel	Major	Monitor closely
Tadalafil – Tamsulosin	Major	Avoid	Excellent	Moderate	Monitor	Fair	Moderate	Adjust dose
Paroxetine - Rivaroxaban	Major	Use caution/Monitor	Fair	Moderate	Monitor	Good	Moderate	Monitor
Meloxicam - Rivaroxaban	Major	Monitor	Fair	Moderate	Monitor	Excellent	Major	Monito closely
